# Who is the best haploidentical donor for acquired severe aplastic anemia? Experience from a multicenter study

**DOI:** 10.1186/s13045-019-0775-9

**Published:** 2019-09-02

**Authors:** Lan-Ping Xu, Shun-Qing Wang, Yan-Ru Ma, Su-Jun Gao, Yi-Fei Cheng, Yuan-Yuan Zhang, Wen-Jian Mo, Xiao-Dong Mo, Yu-Ping Zhang, Chen-Hua Yan, Yu-Hong Chen, Ming Zhou, Yu Wang, Xiao-Hui Zhang, Kai-Yan Liu, Xiao-Jun Huang

**Affiliations:** 1 National Clinical Research Center for Hematologic Disease, Peking University Institute of Hematology, Peking University People’s Hospital, Beijing, China; 2 Department of Hematology, Guangzhou First People’s Hospital, School of Medicine, South China University of Technology, Guangzhou, China; 3 The First Hospital of Jilin University, Changchun, China; 4 Beijing Key Laboratory of Hematopoietic Stem Cell Transplantation, Beijing, China; 5 Peking-Tsinghua Center for Life Sciences, Beijing, China

**Keywords:** Haploidentical transplantation, Donor selection, Acquired severe aplastic anemia

## Abstract

**Background:**

Haploidentical transplantation has been proposed as an effective treatment for severe aplastic anemia (SAA). The majority of patients have more than one HLA-haploidentical donor. Herein, we compared the outcomes between different donor-recipient relationships for optimal haploidentical donor selection in acquired SAA.

**Methods:**

We conducted a multicenter study based on a registered database of 392 patients with SAA treated with allogeneic hematopoietic stem cell transplantation (allo-HSCT) between 2006 and 2018. In total, 223 patients received grafts from father donors, 47 from mother donors, 91 from siblings, 29 from children, and 2 from collateral donors.

**Results:**

Of the 381 patients who survived more than 28 days, 379 (99.5%) recipients were engrafted. The 2-year overall survival (OS) was 86.6 ± 2.5%, 87.1 ± 4.9%, 84.3 ± 3.9%, and 92.2 ± 5.1% for recipients of father, mother, sibling, and child grafts, respectively, (*P* = 0.706). The 2-year failure-free survival (FFS) was 82.8 ± 2.7%, 86.7 ± 5.1%, 80.8 ± 4.2%, and 92.5 ± 5.1% for recipients of father, mother, sibling, and child grafts, respectively, (*P* = 0.508). There was no difference in the incidence of either acute or chronic graft-versus-host disease (GVHD) among the different donor sources in multivariate analyses. There were also no differences in the OS or FFS among the different donor sources in the Cox regression analysis. However, OS was significantly better in the patients with a shorter history of aplastic anemia (< 12 months), better performance status (ECOG scores 0–1), or moderate graft mononuclear cell (MNC) counts (6–10 × 10^8^/kg), and in female recipients with male donors. The FFS was also higher in patients with a shorter history of aplastic anemia (< 12 months) and better performance status (ECOG scores 0–1).

**Conclusions:**

Fathers, mothers, siblings, and children are all suitable haploidentical donors for patients with SAA.

## Background

Severe aplastic anemia (SAA) is a life-threatening hematological disease characterized by an immune-mediated disorder of hematopoietic stem cells. Allogeneic hematological stem cell transplantation (HSCT) is recommended as the first-line treatment in young patients with an available matched sibling donor (MSD) and as the second-line treatment in older patients who failed immunosuppressive therapy (IST) [1, 2].

However, a significant number of patients requiring urgent HSCT lack an MSD. Moreover, the long-term effects of IST are far from satisfactory due to late sequelae, including relapse and the evolution to clonal diseases.

Under this condition, haploidentical donors (HIDs) have increasingly been proposed as an alternative in the absence of an MSD or a matched unrelated donor, and satisfactory outcomes after haploidentical HSCT have been recently reported [3–10]. The overall survival (OS) rate for HSCT with HIDs ranges from 67.1% [5] to 89.0% [8–10], which is similar with that for MSD transplantation, indicating that they are equally effective [8].

Nearly all patients have at least one HLA-haploidentical related donor, and the selection algorithm of a haploidentical donor for hematological malignancies has been recommended [11–13]. Young, male, and noninherited maternal antigen (NIMA)-mismatched donors were associated with the best survival [11]. Optimal HID selection is essential in providing the recipients the best opportunity of a good outcome, but the best donor for SAA remains undefined. A few studies on HLA-identical HSCT for aplastic anemia have shown that male recipients of transplants from female grafts showed decreased survival compared with other donor-recipient sex matches [3, 14, 15]. In addition, for unrelated donors, older donor age (≥ 35 years or ≥ 40 years) is an adverse factor [14, 16]. Thus, this study aimed to identify the best haploidentical donor for acquired severe aplastic anemia. Towards this goal, we compared the outcomes between different donor-recipient relationships.

## Methods

### Patients and study design

This multicenter study was conducted based on a registered database. We included patients with SAA who received a haploidentical transplant between 2006 and 2018 and who received standard busulfan (BU)/cyclophosphamide (CY) conditioning at Peking University Institute of Hematology (*n* = 274), Guangzhou First People’s Hospital (*n* = 92), and the First Affiliated Hospital of Jilin University (*n* = 26). In total, 392 patients were enrolled in the study. The study protocol was approved by the institutional review board. All of the patients gave their written informed consent for the procedure.

### HLA typing

Donor and recipient HLA-A, HLA-B, and HLA-DR1 were performed using high-resolution DNA techniques. All reagents (Special Monoclonal Tray-Asian HLA Class I and Micro SSP HLA Class I and II ABDR DNA Typing Tray; One Lambda, Canoga Park, CA) were approved by the FDA and commercially imported [17].

### Transplant protocols

The transplantation procedure has been described in previous studies [8–10]. The uniform conditioning regimen consisted of busulfan (BU; 3.2 mg/kg/d, intravenous, days − 7 to − 6), cyclophosphamide (CY; 50 mg/kg, days − 5 and − 2), and rabbit anti-thymocyte globulin (ATG; 2.5 mg/kg/d, days − 5 to − 2). Bone marrow (BM) grafts were collected on day 1. Peripheral blood stem cells (PBSCs) were collected via apheresis using a COBE Blood Cell Separator (Gambro BCT, Lakewood, CO, USA) on day 2. All of the transplantation recipients received cyclosporine A (CsA), mycophenolate mofetil (MMF), and short-term methotrexate (MTX) as graft-versus-host disease (GVHD) prophylaxis. Intravenous CsA was administered starting at day − 9 at a dose of 1.5 mg/kg q12h (trough level 200–250 ng/ml) in combination with of MTX (15 mg/m2 day + 1, 10 mg/m^2^ on days + 3, + 6, + 11). CsA was given orally once patients’ bowel function returned to normal. The target concentration of CsA was required within a year posttransplant and was tapered and discontinued over the following 2–3 months. In addition, all patients began MMF orally (500 mg for adults and 250 mg for children, q12h) on day − 9, tapered on day + 30, and discontinued on day + 60 [9]. The recipients received 5 μg/kg subcutaneous G-CSF daily from day + 6 until myeloid recovery. As previously described, all patients were hospitalized in a laminar airflow room and received prophylactic antibiotics during the neutropenic phase [18, 19].

### Definitions

Neutrophil recovery was defined as the first of 3 consecutive days that an absolute neutrophil count ≥ 0.5 × 10^9^/L was achieved, and platelet recovery was defined as the first of 7 consecutive days that a platelet count ≥ 20 × 10^9^/L without transfusion was achieved. Donor recipient chimerism was confirmed by fluorescence in situ hybridization (FISH) for donor/recipient sex-mismatched pairs and by multiplex short tandem repeat (STR) polymerase chain reaction (PCR) for donor/recipient sex-matched pairs. Primary graft failure was defined when the neutrophil counts did not exceed 0.5 × 10^9^/L for 3 consecutive days with low/absent donor chimerism at day 28 post-HSCT. Secondary graft failure or graft rejection was defined as neutropenia (< 0.5 × 10^9^/L) with low/absent donor chimerism in patients with a prior history of engraftment. Patients with neutropenia (< 0.5 × 10^9^/L) but complete donor chimerism were considered to have poor graft function (PGF) [20]. OS was calculated from the date of HSCT to death from any cause. Failure-free survival (FFS) was calculated from the date of HSCT to primary graft failure, secondary graft failure, PGF, second HSCT, or death [21, 22], with the patients alive at the last follow-up administratively censored. GVHD-free/relapse-free survival (GRFS) was calculated from the date of HSCT to the date of events that included grades III–IV acute GVHD, chronic GVHD requiring systemic therapy, relapse, or death [23]. The severity of acute GVHD and chronic GVHD was evaluated according to international criteria [24, 25].

### Statistical analysis

The last follow-up date was February 1, 2019. The endpoint for the study was OS and FFS. The Kruskal-Wallis rank sum test was used for continuous variables, and the chi-square test or Fisher’s exact test was used for categorical variables. All tests were two-sided. The Kaplan-Meier outcome curves for the OS, FFS, and GRFS were constructed for the 392 patients. The log-rank test was used to identify prognostic factors, and a Cox proportional hazards regression model was used to assess the relative impact of previously defined risk factors in multivariate analysis. The cumulative incidences of graft failure and GVHD were calculated in a competing risk model, with death as the competing event. All factors with *P* < 0.10 in univariate analysis along with the donor source were included in multivariate regression. A *P* < 0.05 was considered significant. The data analyses were conducted primarily with SPSS software (SPSS, Chicago, IL, USA), and R software (version 2.6.1) (http://www.r-project.org).

## Results

### Basic characteristics

Table 1 shows the patient, disease, and transplant characteristics of the 392 patients in the study. Also shown in Table 1, the parental donors were older than the sibling donors (father vs. sibling, *P* = 0.000; mother vs. sibling, *P* = 0.000), and the sibling donors were older than the child donors (*P* = 0.007). There was a higher male/female ratio of recipients in the sibling donor group, compared to the parental donor groups (father-child vs. sibling-sibling group, *P* = 0.010; mother-child vs. sibling-sibling group, *P* = 0.020). The history of IST, the interval between diagnosis and transplant, and the infused cell number were similar among the different donor-recipient relationship groups. However, the median CD34+ cells were lower in the mother donor group than in the sibling donor group (*P* = 0.019) and the child donor group (*P* = 0.020).

**Table 1:** Patient, disease, and transplant characteristics

Characteristics	Donor source				
	Father (*N* = 223)	Mother (*N* = 47)	Sibling (*N* = 91)	Child (*N* = 29)	*P*
Patient age					
Median (range), years	12 (1–36)	11 (4–30)	27 (4–55)	43 (29–54)	0.000
≥ 20 years	61 (27.4%)	14 (29.8%)	72 (79.1%)	29 (100%)	0.000
< 20 years	162 (72.6%)	33 (70.2%)	19 (20.9%)	0 (0%)	
Patient gender					0.040
Male	119 (53.4%)	23 (48.9%)	63 (69.2%)	18 (62.1%)	
Female	104 (46.6%)	24 (51.1%)	28 (30.8%)	11 (37.9%)	
Donor age					
Median (range), years	41 (24–63)	37 (20–54)	28 (7–53)	16 (10–28)	0.000
≥ 40 years	125 (56.1%)	19 (40.4%)	6 (6.6%)	0 (0%)	0.000
< 40 years	98 (43.9%)	28 (59.6%)	85 (93.4%)	29 (100%)	
Donor gender					0.000
Male	223 (100%)	0 (0%)	54 (59.3%)	19 (65.5%)	
Female	0 (0%)	47 (100%)	37 (40.7%)	10 (34.5%)	
Donor-recipient gender					0.000
Male to male	119 (53.4%)	0 (0%)	40 (44.0%)	11 (37.9%)	
Female to male	0 (0%)	23 (48.9%)	23 (25.3%)	7 (24.1%)	
Male to female	104 (46.6%)	0 (0%)	14 (15.4%)	8 (27.6%)	
Female to female	0 (0%)	24 (51.1%)	14 (15.4%)	3 (10.3%)	
Months from diagnosis to transplant					
Median (range), months	12 (1–260)	8 (1–144)	12 (1–468)	13 (1–264)	0.374
< 12 months	108 (48.4%)	28 (59.6%)	43 (47.3%)	11 (37.9%)	0.301
≥ 12 months	115 (51.6%)	19 (40.4%)	48 (52.7%)	18 (62.1%)	
Transfusion before transplant					
RBC, median (range), U	20 (1–600)	17 (0–180)	20 (2–300)	16 (2–160)	0.724
PLT, median (range), U	13.5 (0–248)	11.5 (0–60)	16 (0–80)	15 (2–120)	0.721
Ferritin					
Median (range), ng/ml	1701.5 (9–20251)	1801 (189–7434)	1978.5 (23–10550)	1904 (197–8467)	0.633
ECOG score pre-SCT, median (range)					0.406
0	55 (24.7%)	7 (14.9%)	17 (18.7%)	6 (20.7%)	
1	121 (54.3%)	26 (55.3%)	57 (62.6%)	19 (65.5%)	
≥ 2	47 (21.1%)	14 (29.8%)	17 (18.7%)	4 (13.8%)	
Previous ATG treatment					0.211
Yes	42 (18.8%)	8 (17.0%)	14 (15.6%)	1 (3.4%)	
No	181 (81.2%)	39 (83.0%)	77 (84.6%)	28 (96.6%)	
Matched HLA loci at A, B, DR					0.032
3/6	188 (84.3%)	29 (61.7%)	68 (74.7%)	23 (79.3%)	
4/6	26 (11.7%)	13 (27.7%)	18 (19.8%)	5 (17.2%)	
5–6/6	9 (4.0%)	5 (10.6%)	5 (5.5%)	1 (3.4%)	
Donor-recipient ABO match status					0.326
Match	124 (55.6%)	23 (48.9%)	46 (50.5%)	20 (69.0%)	
Minor mismatch	47 (21.1%)	14 (29.8%)	15 (16.5%)	4 (13.8%)	
Major mismatch	40 (17.9%)	8 (17.0%)	20 (22.0%)	3 (10.3%)	
Bidirectional mismatch	12 (5.4%)	2 (4.3%)	10 (11.0%)	2 (6.9%)	
Number of nucleated cells					
Median (range), × 10^8^/kg	9.44 (5.07–25.13)	9.18 (6.57–15.29)	9.93 (4.06–18.11)	9.88 (5.82–16.86)	0.628
Number of CD34-positive cells					
Median (range), × 10^6^/kg	3.12 (0.14–22.47)	2.52 (0.67–10.31)	3.26 (0.57–11.48)	3.48 (0.84–8.72)	0.045

*ATG* anti-thymocyte globulin, *ECOG* Eastern Cooperative Oncology Group, *PLT* platelet, *RBC* red blood cell, *SCT* stem cell transplantation.

### Engraftment

Of the 381 patients who survived more than 28 days, 379 (99.5%) achieved myeloid engraftment at a median time of 12 days (range, 9–31 days), and 365 patients (95.8%) had platelet engraftment at a median time of 14 days (range, 5–180 days). One patient failed engraftment from father donor transplantation, and she had full chimerism after a second transplant from the mother donor. One patient who did not achieve engraftment after a sister donor graft remained in graft failure after a second transplantation from the same donor. This patient eventually underwent transplantation from a matched unrelated donor and achieved hematopoietic recovery. The cumulative incidence of neutrophil recovery at 28 days post-transplant was 96.4 ± 0%, and the cumulative incidence of platelet recovery at 100 days post-transplant was 91.3 ± 0%. Of the nine patients who experienced secondary graft failure (six from father grafts and three from brother grafts), two received a second transplant from the second haplo-donor, and one received an unrelated donor graft. All three patients achieved engraftment, but two of them died from an infection. Two patients who received second transplants from the same donor and another two patients who received donor lymphocyte infusion (DLI) did not achieve engraftment. The remaining two patients did not receive either second transplants or DLI.

Detection of donor-specific antibody (DSA) was performed in 196 of the 392 patients, and 14 patients (7.1%) exhibited positive DSA. The proportions of positive DSA were 6.0% (*n* = 8), 6.7% (*n* = 1), 8.3% (*n* = 3), and 20.0% (*n* = 2) in the father, mother, sibling, and child donor groups, respectively, (*P* = 0.417). Of the 11 patients with graft failure, DSA was measured in four patients, and one patient with primary graft failure had positive DSA.

### GVHD

**Fig. 1:**
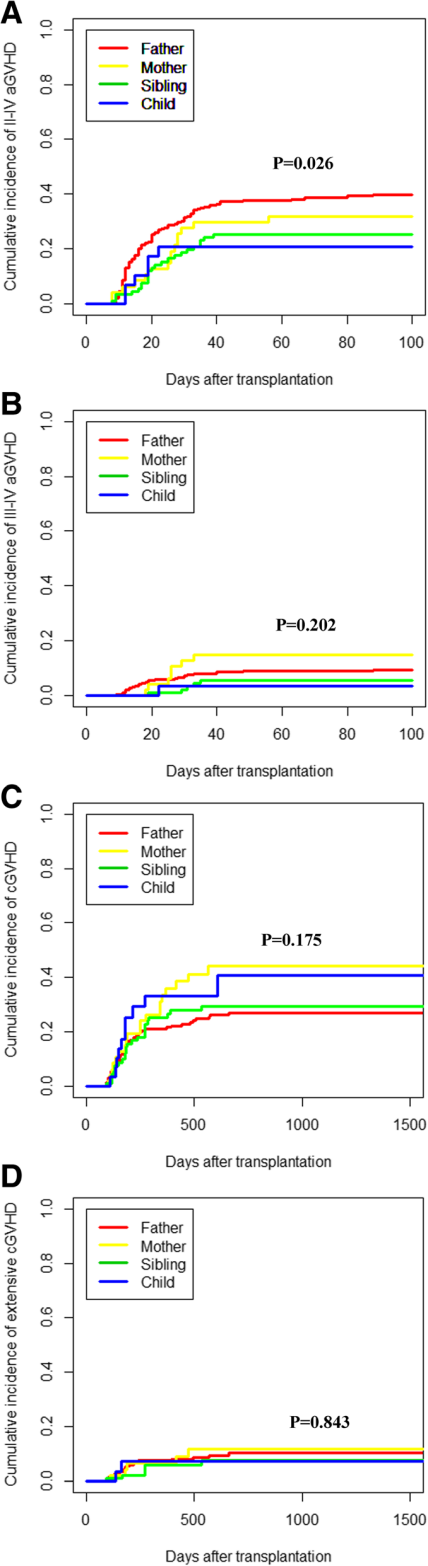
**a** Grade II–IV aGVHD in different donor kinships. **b** Grade III–IV aGVHD in different donor kinships. **c** cGVHD in different donor kinships. **d** Extensive cGVHD in different donor kinships

The cumulative incidence of grade II–IV aGVHD at 100 days was lower among the recipients of sibling grafts than that in parental transplants (*P* = 0.011). In the competing risk model, mother donor transplants had a higher rate of cGVHD (44.3 ± 0.6% vs. 27.1 ± 0.1%, *P* = 0.046) but not aGVHD (31.9 ± 0.5% vs. 39.9 ± 0.1%, *P* = 0.272) compared with father donor transplants (Table 2; Fig. 1). A higher degree of HLA mismatching was associated with a higher risk of grade II–IV aGVHD, but there were no significant differences in the risk of grade III–IV aGVHD and cGVHD. There were also no significant differences between NIMA and noninherited paternal antigen (NIPA) mismatched sibling donors in aGVHD and cGVHD.

**Table 2:** Univariate analysis of donor-related characteristics on transplant outcomes

Risk factors	II–IV aGVHD	III–IV aGVHD	Chronic GVHD	Extensive cGVHD	FFS	OS
Relationship	100d estimated cumulative incidence		2-year estimated cumulative incidence or probability			
Paternal donor	39.9% ± 0.1%	9.4%±0.0%	27.1%±0.1%	10.3%±0.1%	82.8%±2.7%	86.5% ± 2.5%
Maternal donor	31.9% ± 0.5%	14.9% ± 0.3%	44.3% ± 0.6%	11.9% ± 0.3%	86.7% ± 5.1%	87.1% ± 4.9%
Sibling donor	25.3% ± 0.2%	5.5% ± 0.1%	29.5% ± 0.3%	7.5% ± 0.1%	80.8% ± 4.2%	84.3% ± 3.9%
Offspring donor	20.7% ± 0.6%	3.4% ± 0.1%	40.6% ± 1.2%	7.2% ± 0.2%	92.5% ± 5.1%	92.5% ± 5.1%
*P*	0.026	0.202	0.175	0.843	0.508	0.706
Paternal donor	39.9% ± 0.1%	9.4% ± 0.0%	27.1% ± 0.1%	10.3% ± 0.1%	82.8% ± 2.7%	86.5% ± 2.5%
Maternal donor	31.9% ± 0.5%	14.9% ± 0.3%	44.3% ± 0.6%	11.9% ± 0.3%	86.7% ± 5.1%	87.1% ± 4.9%
*P*	0.272	0.282	0.046	0.780	0.937	0.755
Sibling donor	25.3% ± 0.2%	5.5% ± 0.1%	29.5% ± 0.3%	7.5% ± 0.1%	80.8% ± 4.2%	84.3% ± 3.9%
Offspring donor	20.7% ± 0.6%	3.4% ± 0.1%	40.6% ± 1.2%	7.2% ± 0.2%	92.5% ± 5.1%	92.5% ± 5.1%
*P*	0.697	0.670	0.333	0.858	0.130	0.252
Paternal donor	39.9% ± 0.1%	9.4% ± 0.0%	27.1% ± 0.1%	10.3% ± 0.1%	82.8% ± 2.7%	86.5% ± 2.5%
Sibling donor	25.3% ± 0.2%	5.5% ± 0.1%	29.5% ± 0.3%	7.5% ± 0.1%	80.8% ± 4.2%	84.3% ± 3.9%
*P*	0.011	0.238	0.699	0.445	0.478	0.475
Donor age						
< 30	27.2% ± 0.2%	4.9% ± 0.0%	35.0% ± 0.3%	9.2% ± 0.1%	85.8% ± 3.5%	88.2% ± 3.2%
≥ 30	36.3% ± 0.1%	10.0% ± 0.0%	29.1% ± 0.1%	9.7% ± 0.0%	82.3% ± 2.4%	85.6% ± 2.2%
*P*	0.104	0.106	0.194	0.815	0.538	0.659
< 40	32.0% ± 0.1%	7.5% ± 0.0%	28.9% ± 0.1%	7.5% ± 0.0%	86.1% ± 2.3%	88.4% ± 2.1%
≥ 40	37.1% ± 0.2%	10.6% ± 0.1%	32.9% ± 0.2%	12.5% ± 0.1%	79.0% ± 3.5%	83.4% ± 3.2%
*P*	0.383	0.303	0.463	0.162	0.104	0.187
Donor sex						
Male	35.9% ± 0.1%	8.1% ± 0.0%	28.3% ± 0.1%	9.1% ± 0.0%	82.9% ± 2.3%	86.0% ± 2.2%
Female	27.7% ± 0.2%	10.6% ± 0.1%	37.8% ± 0.3%	10.8% ± 0.1%	84.3% ± 3.9%	87.1% ± 3.5%
*P*	0.137	0.459	0.144	0.666	0.901	0.871
Donor-recipient sex						
Male-male	36.0% ± 0.1%	7.0% ± 0.0%	27.2% ± 0.1%	9.7% ± 0.1%	79.0% ± 3.3%	81.4% ± 3.2%
Female-male	22.6% ± 0.3%	9.4% ± 0.2%	40.3% ± 0.6%	17.2% ± 0.3%	87.4% ± 4.9%	92.3% ± 3.7%
Male-female	35.7% ± 0.2%	9.5% ± 0.1%	30.0% ± 0.2%	8.6% ± 0.1%	88.2% ± 3.0%	92.1% ± 2.5%
Female-female	34.1% ± 0.6%	12.2% ± 0.3%	34.7% ± 0.7%	2.6% ± 0.1%	80.5 ± 6.2%	80.3% ± 6.2%
*P*	0.324	0.711	0.459	0.175	0.251	0.030
HLA match						
3/6	36.8% ± 0.1%	5.0% ± 0.3%	28.9% ± 0.1%	7.6% ± 0.0%	83.7% ± 2.2%	86.8% ± 2.0%
4/6	27.4% ± 0.3%	6.5% ± 0.1%	31.7% ± 0.4%	16.4% ± 0.3%	79.3% ± 5.8%	82.6% ± 5.1%
5-6/6	10.0% ± 0.5%	9.4% ± 0.0%	54.5% ± 1.7%	17.5% ± 0.9%	88.2% ± 8.0%	89.1% ± 7.3%
*P*	0.019	0.616	0.307	0.096	0.578	0.551
ABO blood type						
Match	30.8% ± 0.1%	4.7% ± 0.0%	31.4% ± 0.1%	8.5% ± 0.0%	82.1% ± 2.8%	86.5% ± 2.5%
Minor mismatch	42.0% ± 0.3%	17.3% ± 0.2%	32.7% ± 0.3%	9.9% ± 0.1%	84.7% ± 4.1%	87.4% ± 3.7%
Major mismatch	32.3% ± 0.3%	9.9% ± 0.1%	20.7% ± 0.3%	10.0% ± 0.2%	87.8% ± 4.1%	87.8% ± 4.1%
Bidirectional mismatch	38.5% ± 1.0%	11.5% ± 0.4%	43.5% ± 1.1%	15.6% ± 0.5%	75.7% ± 8.8%	77.0% ± 8.3%
*P*	0.415	0.007	0.162	0.668	0.490	0.517

*aGVHD* acute graft-versus-host disease, *cGVHD* chronic graft-versus-host disease, *FFS* failure-free survival, *OS* overall survival.

In the multivariate analysis, patient age < 20 years (vs. ≥ 20 years, HR = 1.863, 95% CI 1.300–2.668, *P* = 0.001) and a higher degree of HLA mismatches (3/6 vs. 5–6/6, HR = 4.702, 95% CI 1.161–19.037, *P* = 0.030) were risk factors for grade II–IV aGVHD. Patient age < 20 years (vs. ≥ 20 years, HR = 2.316, 95% CI 1.081–4.962, *P* = 0.031) and ABO minor mismatch (vs. others, HR = 2.817, 95% CI 1.438–5.519, *P* = 0.003) remained significant prognostic factors for grade III–IV aGVHD in the multivariate model (Table 3).

**Table 3:** Risk factors for survival and GVHD: Cox regression analysis

Risk factor	Relative risk	95% CI	*P* value
II–IV aGVHD			
Patient age < 20 years vs. ≥ 20 years	1.863	1.300–2.668	0.001
HLA 3/6 vs. 5–6/6	4.702	1.161–19.037	0.030
HLA 3/6 vs. 4/6	1.509	0.906–2.512	0.114
III–IV aGVHD			
Patient age < 20 years vs. ≥ 20 years	2.316	1.081–4.962	0.031
ABO minor mismatch vs. others	2.817	1.438–5.519	0.003
cGVHD			
Mother donor vs. father donor	1.804	1.069–3.044	0.027
Overall survival			
Male to female vs. others	0.433	0.210–0.889	0.023
Months from diagnosis to transplant ≥ 12 vs. < 12	1.876	1.057–3.328	0.032
ECOG 2–3 vs. 0–1	3.605	2.028–6.409	0.000
MNC 6–10 × 10^8^/kg vs. others	0.440	0.249–0.778	0.005
Failure-free survival			
Months from diagnosis to transplant ≥ 12 vs. < 12	1.907	1.124–3.235	0.017
ECOG 2-3 vs. 0-1	2.388	1.414–4.032	0.001
GVHD-free/relapse-free survival			
ABO minor mismatch vs. others	1.856	1.245–2.765	0.002
ECOG 2–3 vs. 0–1	1.589	1.016–2.486	0.043
MNC 6–10 × 10^8^/kg vs. others	0.642	0.433–0.953	0.028

*aGVHD* acute graft-versus-host disease, *cGVHD* chronic graft-versus-host disease, *ECOG* Eastern Cooperative Oncology Group, *MNC* mononuclear cell

Mother donor was the only risk factor for cGVHD in the Cox model (mother donor vs. father donor, HR = 1.804, 95% CI 1.069–3.044, *P* = 0.027) (Table 3). The Cox model did not reveal significant differences in cGVHD when the mother donors were compared to the sibling (HR = 1.496, 95% CI 0.824–2.717, *P* = 0.186) or child donors (HR = 1.158, 95% CI 0.538–2.492, *P* = 0.707). No significant factors in extensive cGVHD were identified in the multivariate analysis.

### Infection

Of the 392 SAA patients, 298 (76.0%) had virus infections, 85 (21.7%) had bacterial infections, and 33 (8.4%) had fungal infections. Cytomegalovirus (CMV) antigenemia occurred in 284 patients (72.4%), and eight patients developed CMV diseases (four pneumonia, two enteritis, and two retinitis). Epstein-Barr virus (EBV) antigenemia occurred in 56 patients (14.3%), and eight developed post-transplant lymphoproliferative disorders. No cases of adenovirus infection and toxoplasma were observed.

### Survival

**Fig. 2:**
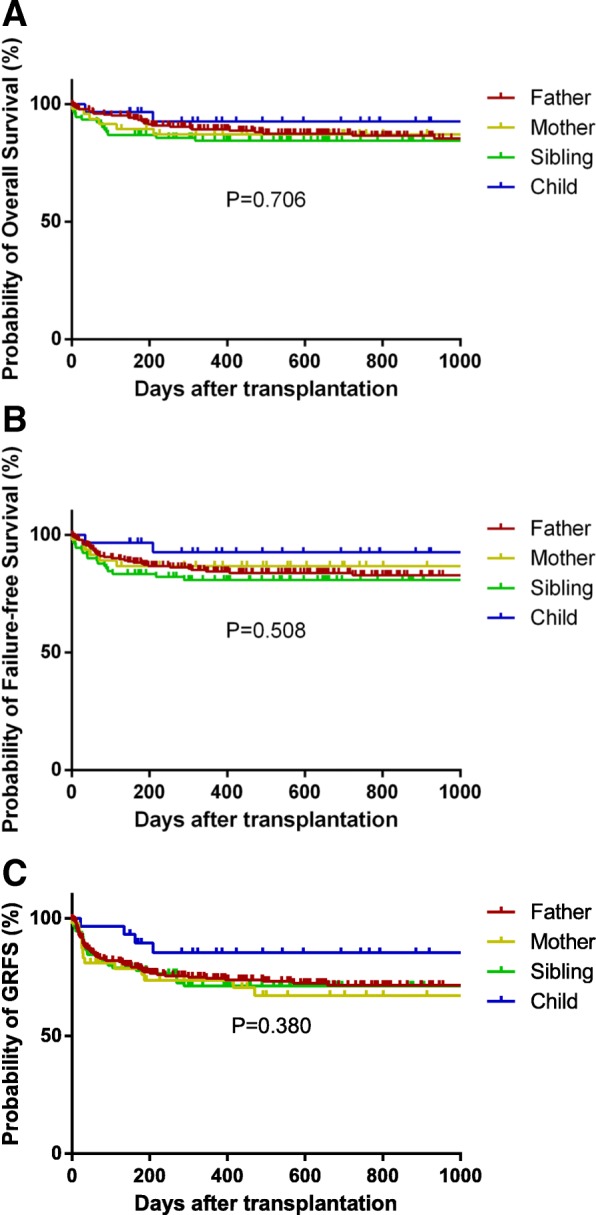
**a** The 2-year overall survival of different donor kinships: father donor, 86.6 ± 2.5%; mother donor, 87.1 ± 4.9%; sibling donor, 84.3 ± 3.9%; child donor, 92.2 ± 5.1% (*P* = 0.706)**. b** The 2-year failure-free survival of different donor kinships: father donor, 82.8 ± 2.7%; mother donor, 86.7 ± 5.1%; sibling donor, 80.8 ± 4.2%; child donor, 92.5 ± 5.1% (*P* = 0.508)**. c** The 2-year GVHD-free/relapse-free survival of different donor kinships: father donor, 71.5 ± 3.2%; mother donor, 67.1 ± 7.4%; sibling donor, 71.1 ± 4.9%; child donor, 85.3 ± 6.8% (*P* = 0.380)

In total, 52 (13.3%) patients died. The causes of death were GVHD in seven cases, infection in 20 cases, and other complications (in 25 cases). At a median follow-up of 743 days (range 125–4754 days), the OS, FFS, and GRFS were both similar among the recipients from different donor kinships. At last follow-up, 195 patients (87.4%) in the father donor group, 40 (85.1%) in the mother donor group, 77 (84.6%) in the sibling donor group, 27 (93.1%) in the child donor group, and one (50%) in the collateral donor group were alive. The long-term outcomes of the patients are shown in Fig. 2.

In the Cox regression analysis, there were no differences in the OS and FFS according to the donor graft source, but both the OS and FFS were significantly better in the patients with a short history of aplastic anemia (≥ 12 vs. < 12 months, HR = 1.876, 95% CI 1.057–3.328, *P* = 0.032), and better performance status (ECOG scores 2–3 vs. 0–1, HR = 3.605, 95% CI 2.028–6.409, *P* = 0.000). The 2-year OS rate of the male-to-female transplants was higher than that of other sex-matched groups (92.1 ± 2.5% vs. 83.5 ± 2.4%) in the Kaplan-Meier survival curve, and male-to-female donations remained a significantly beneficial prognostic factor for the OS in the multivariate analysis (HR = 0.433, 95% CI 0.210–0.889, *P* = 0.023). Moderate MNC counts of 6–10 × 10^8^/kg were also a protective factor against death (for OS, HR = 0.440, 95% CI 0.249–0.778, *P* = 0.005). The covariates that were significant for GRFS included ABO minor mismatch, MNC counts, and ECOG score (Table 3).

There were no statistically significant differences in OS or FFS between NIMA- and NIPA-mismatched sibling donor transplants (OS 80.6 ± 7.1% vs. 83.5 ± 6.2%, *P* = 0.066; FFS 80.6 ± 7.1% vs. 83.4 ± 6.2%, *P* = 0.708).

### Subgroup analysis

In the subgroup analysis, for the female recipients, female donors were associated with worse OS than male donors in the multivariate analysis (HR = 2.581, 95% CI 0.990–6.728, *P* = 0.052). In contrast, female donors were not associated with worse survival when the recipient was male (male donors vs. female donors: 2-year OS, 81.4 ± 3.2% vs. 92.3 ± 3.7%, *P* = 0.150; 2-year FFS, 79.0 ± 3.3% vs. 87.4 ± 4.9%, *P* = 0.387, respectively).

In total, 29 patients received child grafts (19 from sons and 10 from daughters). There were no differences in OS, FFS, and GVHD between son donor and daughter donors (2-year OS: 88.4 ± 7.8% vs. 100%, *P* = 0.289; 2-year FFS: 88.4 ± 7.8% vs. 100%, *P* = 0.289; grade II–IV aGVHD: 26.3% vs. 10.0%, *P* = 0.301; grade III–IV aGVHD: 5.3% vs. 0%, *P* = 0.468; cGVHD: 48.1% vs. 32.5%, *P* = 0.503; extensive cGVHD: 5.6% vs. 10.0%, *P* = 0.723).

There were 91 patients who received sibling donor grafts. Regarding male donor to male patient (M-M), female donor to male patient (F-M), male donor to female patient (M-F), and female donor to female patient (F-F), the 2-year OS was 82.1 ± 6.2%, 90.9 ± 6.2%, 92.9 ± 6.9%, and 71.4 ± 12.1% (*P* = 0.308), respectively. The 2-year FFS for M-M, F-M, M-F, and F-F was 82.5 ± 6.0%, 80.2 ± 9.0%, 84.6 ± 10.0%, and 71.4 ± 12.1% (*P* = 0.773), respectively. There were no differences in either aGVHD or cGVHD between different donor-recipient sex matches.

For pediatric patients, the Cox models for OS and FFS revealed a significant influence of better performance status (ECOG scores 2–3 vs. 0–1; OS: HR = 5.312, 95% CI 2.092–13.487, *P* = 0.000; FFS: HR = 3.547, 95% CI 1.585–7.937, *P* = 0.002). Donor-to-recipient sex match, the duration from diagnosis to transplant, and MNC counts did not affect OS and FFS for pediatrics. ABO minor mismatch remained a significant risk factor for grade III–IV aGVHD (ABO minor mismatch vs. others, HR = 3.467, 95% CI 1.520–7.909, *P* = 0.003). No significant clinical factors were found to be associated with grade II–IV aGVHD, cGVHD, and extensive cGVHD.

For adult patients, the significant covariates for OS were better performance status (ECOG scores 2–3 vs. 0–1, HR = 3.473, 95% CI 1.610–7.489, *P* = 0.001), and moderate MNC counts of 6–10 × 10^8^/kg (HR = 0.282, 95% CI 0.131–0.608, *P* = 0.001). These two factors were also significant in the Cox model for FFS (ECOG scores 2–3 vs. 0–1, HR = 2.627, 95% CI 1.247–5.534, *P* = 0.011; moderate MNC counts of 6–10 × 10^8^/kg vs. others, HR = 0.345, 95% CI 0.169–0.704, *P* = 0.003). Meanwhile, we did not find any statistically significant factors for GVHD.

## Discussion

In this study, we compared the outcomes between different donor-recipient relationships to help prioritize haploidentical donors for SAA to improve survival after haploidentical transplantation.

We found that maternal grafts were not associated with poorer survival outcomes. Our previous results on leukemia suggested that the survival of transplantation from mother to child was inferior to that from other family relationships in haploidentical HSCT, probably due to the higher probability of GVHD and nonrelapse mortality (NRM) [11]. Wang et al. compared the outcomes of mother donor transplants and contemporaneous transplants using allografts from father donors, and the rate of grade II–IV aGVHD, rate of cGVHD rate, NRM, and survival in the mother donor cohort versus father donor cohort were 52% vs. 40%, 57% vs. 48%, 21% vs. 13%, and 64% vs. 74%, respectively [11]. There are several possible reasons for the inconsistent outcomes between the current and previous studies. First might be the different conditioning regimens between hematological malignancies and aplastic anemia. Cyclophosphamide was a regular approach for immunological tolerance induction in haploidentical HSCT and was given to the SAA patients in this study at a total dose of 200 mg/kg on days − 5 to − 2 [26, 27]. Thus, at a higher drug dose, it seemed plausible that enhanced in vivo graft-host tolerance was induced. Secondly, in recent years, progress in the understanding, prophylaxis, and therapeutic intervention for GVHD has reduced the probability and severity of GVHD [28, 29], and the discovery of predictive and prognostic biomarkers might be helpful for individualized GVHD prophylaxis [30]. Further, the mother donor group had a higher frequency of ABO minor mismatch. Because ABO minor mismatch was related to grade III–IV aGVHD, we also analyzed the underlying factor of blood type that might intensify the association between mother donor and cGVHD. However, no influence of ABO blood type on cGVHD was detected in either univariate or multivariate models. In our cohort, although the mother donor group had a higher incidence of cGVHD than the father donor group, the mother donor group did not demonstrate more frequent cGVHD compared to the sibling or child donor groups, and the rates of aGVHD and extensive cGVHD were similar between the mother donor group and other donor groups.

In haploidentical transplants for hematological malignancies, the number of HLA locus disparities was not significantly correlated with transplant outcomes [11]. However, in this population of haploidentical transplants for SAA, we found transplants with HLA mismatches of 0 or 1 locus had a lower rate of grade II–IV aGVHD than those with three loci HLA mismatches. As mentioned above, the inconsistent outcomes between hematological malignancies and SAA might be due to the different physiology between the two diseases and the different conditioning regimens used.

Our findings are in line with a study showing a higher rate of III–IV aGVHD in the minor ABO-mismatched group in 154 unrelated donor transplants [31]. In 154 cases, Ludajic et al. reported that the cumulative incidence of III–IV aGVHD was 37% in the minor ABO-mismatched group compared to 18% in the ABO-matched group, 10% in the major ABO-mismatched group, and 14% in the bidirectional ABO-mismatched group. Compared to the ABO-matched group, the risk of III–IV aGVHD was approximately 4-fold high in the minor ABO-mismatched group [31]. Ozkurt et al. reported more frequent severe aGVHD among minor ABO-mismatched patients [32], but Kimura et al. found a high incidence of severe aGVHD in both major and minor ABO mismatches [33]. There were also conflicting results that failed to find a difference in rates of aGVHD between minor ABO incompatibility and others [34–36]. No evidence of an association between the ABO mismatch and cGVHD or survival was observed in this study. As the recipient A or B antigens are also expressed on their endothelial and epithelial cells, the mechanism of more severe aGVHD in minor ABO mismatches is likely to be the immunization of donor B lymphocytes against recipient antigens inducing GVHD. Most studies investigating the effect of ABO mismatches on GVHD were based on hematological malignancies and mostly unrelated donor transplants. For SAA patients, we found that minor ABO mismatch was a detrimental factor for severe aGVHD in haploidentical transplantation.

In this SAA population, the OS and FFS were better in patients receiving moderate MNC counts. The target count of MNC was 6–8 × 10^8^/kg in haploidentical allogeneic transplantation for SAA, while in our clinical practice, the MNC count infused ranged from 6 to 10 × 10^8^/kg [9]. In this population, preliminary analysis showed that MNC counts of 6–10 × 10^8^/kg were better than < 6 and > 10 × 10^8^/kg for survival. Thus, we recommended 6–10 × 10^8^/kg as a moderate range of MNC counts. The lower CD34+ cell harvest from mother donors is an interesting phenomenon. Previous studies showed that age, sex, and body mass index (BMI) might be associated with CD34+ cell count yield, but this association is still controversial [37–39]. Our previous study on the impact of donor characteristics on the immune cell composition showed that donor sex was not correlated with the yield of CD34+ stem cells [38]. However, in this study, female donors had lower CD34+ cell counts than men (median, 2.63 × 10^6^/kg vs. 3.33 × 10^6^/kg, *P* = 0.020). In another study, the researchers observed a lower post-G-CSF CD34+ cell count in female donors than in men, and they speculated that this difference might be because female donors weighed less than male donors and received lower total amounts of G-CSF [39]. Chen et al. also reported that female donors were less excellent responders in terms of CD34+ cell count [40]. However, there are currently no data on the relationship between mother donors and CD34+ cell collection and the results need further validation.

Female donation to a male recipient was regarded as an adverse prognostic factor in transplantation for hematological malignancies [41, 42] and in HLA-matched HSCT for SAA [14, 15]. In a multicenter study of aplastic anemia, Stern et al. reported decreased survival and increased risk of rejection in female patients with male donors compared to recipients of sex-matched grafts [15]. By contrast, we observed that female recipients with male donors had better survival than recipients of sex-matched grafts. However, the reason for this finding remains unknown. The importance of donor sex and age for SAA in allogeneic HSCT has been widely studied [14, 15]. Grafts from younger donors have been reported to be a favorable factor for survival after haploidentical transplantation for SAA [14, 16]. However, older donors were not associated with more GVHD or worse survival than younger donors in our study.

A potential limitation of this retrospective study is that based on the current decision-making for donor selection in hematologic malignancies, mother donors are rarely selected. Therefore, improvement in the SAA survival of maternal graft transplantation might be partially underestimated. Even so, the mother donor group still demonstrated comparable outcomes with other donor groups, thus showing that maternal grafts were not inferior to grafts from other donors. Although our data showed that male donors for female recipients might be superior to female donors, this finding was drawn from limited data and is not supported by previous reports. Therefore, the results may not be generalizable to other transplant patterns, and prospective studies are warranted.

## Conclusion

In conclusion, fathers, mothers, siblings, and children are all suitable HIDs for patients with SAA. Despite the higher incidence of cGVHD in mother donors, survival outcomes were comparable for father, mother, sibling, and child donor transplantation. To reduce the risk of severe aGVHD, avoiding donor-recipient ABO blood minor mismatch should be considered. These findings provide a basis for the selection of optimal HIDs for SAA.

## Data Availability

The datasets used and/or analyzed during the current study are available from the corresponding author on reasonable request.

## Abbreviations

aGVHD: Acute graft-versus-host disease
allo-HSCT: Allogeneic hematopoietic stem cell transplantation
ATG: Anti-thymocyte globulin
BM: Bone marrow
BU: Busulfan
cGVHD: Chronic graft-versus-host disease
CsA: Cyclosporin A
CY: Cyclophosphamide
ECOG: Eastern Cooperative Oncology Group
FFS: Failure-free survival
FISH: Fluorescence in situ hybridization
GRFS: GVHD-free/relapse-free survival
HID: Haploidentical donor
HSCT: Hematopoietic stem cell transplantation
IST: Immunosuppressive therapy
MMF: Mycophenolate mofetil
MNC: Mononuclear cell
MSD: Matched sibling donor
MTX: Methotrexate
NIMA: Noninherited maternal antigen
NIPA: Noninherited paternal antigen
NRM: Nonrelapse mortality
OS: Overall survival
PBSCs: Peripheral blood stem cells
PCR: Polymerase chain reaction
PGF: Poor graft function
PTLD: Post-transplant lymphoproliferative disorders
SAA: Severe aplastic anemia
STR: Short tandem repeat
